# Estimating Service Demand for Intermediary Care at a Community Integrated Intermediary Care Center among Family Caregivers of Older Adults Residing in Chiang Mai, Northern Thailand

**DOI:** 10.3390/ijerph18116087

**Published:** 2021-06-04

**Authors:** Thin Nyein Nyein Aung, Myo Nyein Aung, Saiyud Moolphate, Yuka Koyanagi, Mariko Ichikawa, Siripen Supakankunti, Motoyuki Yuasa

**Affiliations:** 1 Department of Public Health, Graduate School of Medicine, Juntendo University, Tokyo 113-8421, Japan; a-thin@juntendo.ac.jp (T.N.N.A.); moyuasa@juntendo.ac.jp (M.Y.); 2 Advanced Research Institute for Health Science, Juntendo University, Bunkyo City, Hongo, 2 Chome-1-1, Tokyo 113-8421, Japan; 3 Faculty of International Liberal Arts, Juntendo University, Tokyo 113-8421, Japan; 4 Department of Public Health, Faculty of Science and Technology, Chiang Mai Rajabhat University, Chiang Mai 50300, Thailand; saiyudmoolphate@gmail.com; 5 Tokyo Ariake University of Medical and Health Sciences, Tokyo 135-0063, Japan; koyanagiy@tau.ac.jp; 6 Yutaka Clinic, 4-18-21 Yutaka-cho, Shinagawa-ku, Tokyo 142-0042, Japan; m.ichikawa.tu@juntendo.ac.jp; 7 Centre of Excellence for Health Economics, Faculty of Economics, Chulalongkorn University, Bangkok 10330, Thailand; Siripen.S@chula.ac.th

**Keywords:** ageing, Anderson’s behavioral model of health care use, Community Integrated Intermediary Care (CIIC), family caregiver burden, global health, intermediary care, long-term care, respite care, TCTR20190412004, Thailand

## Abstract

Background: Thailand’s population is currently the third most rapidly aging in the world, with an estimated 20 million ageing population by 2050. Sustainability of the family based long-term care model is challenged by the chronic burden on family caregivers and by smaller family sizes. We aimed to introduce a new service model, Community Integrated Intermediary Care (CIIC), TCTR20190412004, including free of charge intermediary care services at CIIC centers in the local community, to help older adults whose caregivers are temporarily unable to sustain care at home. Since Thai society upholds values of gratefulness, it is better to estimate willingness to use such an intermediary care service first, before introducing the service. Methods: A total of 867 pairs of senior citizens and their family caregivers were interviewed with structured-questionnaires in 2019. Descriptive analysis and binary logistic regression were applied to determine the predictors of family caregivers’ willingness to use the CIIC service, guided by Anderson’s model of health services use. Results: About 26.8% of elderly participants and 24.0% of family caregivers were willing to use an intermediary care service. The family caregiver determinants of predisposing factors (kinship: spouse caregivers, other relatives, maid or friends; job types: own business and private company staff), enabling factors (original community residents and monthly income ≤9000 baht), and need factors (caregiver burden total scores ≥24, taking leave for caregiving, and having diabetes), were found to be significantly associated with willingness to use the CIIC service. Conclusions: The baseline survey data noted that caregivers’ sociodemographic factors and burden determined their willingness to use the intermediary care service, although the dependency of care recipients was low in this study. This, nonetheless, indicated that there is need for a backup respite care to strengthen current family based long-term aging care in Thailand.

## 1. Introduction

The aging population is rapidly increasing in Thailand and the demographic shift from a younger to an older population age structure began to occur almost two decades ago. By the year 2050, about 64% of dependents will be older adults in Thailand, suggesting higher old age dependency ratios, which in turn would result in a heavier burden of support for these older people [1]. Traditionally, a Family-based Long-Term Care (FLTC) model is practiced whereby elders rely largely on their families to provide material support and care. While Thailand has a strong health system with well-established universal health insurance coverage, it is still in need of Long-Term Care (LTC) insurance and appropriate policies to finance an effective LTC model for the estimated 20 million aging population in 2050 [2,3]. Almost all the elder persons who need LTC receive informal care provided by their families and relatives [4,5,6]. These informal caregivers can incur severe strains on their physical and mental health, not only due to the chronic burden of caregiving but also due to the reduction in family sizes and the migration of adult children for job opportunities [7,8,9,10]. While some residential homes are financed by the government and charitable organizations, most of the residential long-term care services for dependent older persons are provided by private nursing homes and private hospitals. Statistics for the number of existing residential LTC institutions and the need for human resources cannot be estimated as there is no LTC information system. The current policy agenda does not include public financing and provision of residential care [11]. Therefore, there is an apparent need for the government to create evidence-based policies and programs to meet the needs of this new aging demographic and to strengthen the traditional FLTC model. 

To address this issue, a cluster randomized control trial (Community Integrated Intermediary Care (CIIC) project), consisting of six control clusters and six intervention clusters, with a new respite care service, will be introduced among the intervention arms [12]. Intermediary or respite care is formal care which is neither day care nor long-term residential care, provided for short-term relief of burdened caregivers when they are not temporarily available or are suffering from burnout. This service will be provided at the newly established CIIC center, located in the community, staffed with professional health care personnel and volunteers. It is a totally free of charge service and the maximum length of stay is 10–14 days. Short stay usage can be a preventative and cost-effective measure against family burden as it enables old people to stay at home longer and delays residential care admission [13,14,15,16]. Furthermore, in the context of limited facilities for long term care and the growing number of aging people, the intermediary care model is an attractive option. However, in Thai culture, due to assumptions related to social norms such as filial obligation and reciprocal exchanges for the support of older persons by family members, sending them to an intermediary care facility can be regarded as ignoring their parents. As a result, the willingness of family caregivers to send their older adults to a short stay at CIIC, and care recipients’ desire to use such a residential respite care service, have to be explored first. This cross-sectional survey, using Anderson’s behavioral model of health service use, explored the baseline data of the intervention clusters before the intervention was launched.

According to Anderson, people’s use of health services is determined by societal factors, health service system factors, and individual factors. Individual factors were categorized as a function of their predisposition to use services, enabling or impeding factors and their need for care. Predisposing characteristics influence decision making regarding planned or intended behavior. Enabling factors relate to accessible community and individual level resources. Availability or supply of services and ability to pay or access support funds can impact the utilization of health care services overall. Need factors include individual perceived health, general state of health and functional needs. This concept has been applied to study health service utilization of various types, and attitudes toward such services [17,18,19,20]. With willingness to use a CIIC intermediary care service as an outcome, individual determinants such as predisposing factors (age, gender, marital status, the relationship between the older person and the caregiver, education and occupation), enabling factors (income and type of residence) and potential need factors (care recipients’ demands, family caregiver burden, taking leave for caregiving, physical health status) will be evaluated.

Accessible physical environments are crucial for active aging [21] and the CIIC short stay center is an enabling resource as it will be situated within geographical proximity of participating families’ residence and integrated within the community. As the introduction of respite care service is still new to Northern Thailand, exploration of such data can be anticipated to contribute to the development of an evidence-based approach for family caregivers, providing insights about the caregivers and care recipients who are in potential need of intermediary care services.

## 2. Materials and Methods

### 2.1. Data Collection and Participants

This study was conducted in accordance with the Declaration of Helsinki. The World Health Organization Ethical Review Committee: WHO/ERC ID; ERC.0003064, dated 7 March 2019 and Ethical Review Committee for Research in Human Subjects: Boromarajonani College of Nursing Nakhon Lampang: Praboromarajchnok, Institute for Health Workforce Development, Ministry of Public Health, Thailand (approval number E 2562/005, dated 4 March 2019) approved the ethics of the study. It has been registered at the Thailand Clinical Trial Registry, Trial registration number TCTR20190412004.

This was a subgroup analysis of the participants from an intervention arm of a cluster randomized control trial (CIIC project) before launching the intervention. The study site was in Chiangmai with ageing people comprising 18.2% of the total population, culturally similar to its neighboring country Myanmar in terms of caregiving for older parents as a family tradition and a social value. Co-residence or living in close proximity is a predominant pattern of living arrangements for older adults in both countries. Cultural contexts are relatively similar as the majority of population adhere to Buddhism which recommends values and traditions relating to taking care of older adults as social norms. Children’s filial respect and moral obligation to support and care for their parents prevails. At the same time, older adults believe that they are owed such care as repayment for having nurtured their children. Society also criticizes children who neglect their parents or behave improperly to them [11,22,23].

The CIIC project comprised of six intervention clusters and six control clusters that aimed to recruit 2000 participants in each arm by using STATA version 11SE (Stata Corporation, College Station, TX, USA) for sample size calculation and power estimations. The study participants from Maehia subdistrict, Mueang Chiang Mai, were selected for inclusion in an intervention arm by cluster randomization. Inclusion criteria were persons aged 60 years and above and their family caregivers, either male or female, and residents in the study’s location. Those who did not consent, who could not understand the explanation regarding informed consent, and with cognitive impairment or severe impairment in decision making abilities were excluded. The research assistants were trained in data collection and data were collected according to the CIIC study protocol via interviewer administered survey questionnaires in 2019 [12].

Family caregivers were classified according to the amount of care they provided. Primary caregivers were those who devoted the highest amount of caring. Secondary caregivers were helpers of primary caregivers who supported care for the elderly at home [24]. About 1509 older adults and 875 primary caregivers were recruited in the intervention arm initially, but eight cases (0.91%) of caregivers had incomplete data regarding the main outcome variables of willingness to stay at a CIIC center and incomplete Caregiver Burden Inventory scoring. Therefore, the remaining 867 cases with complete data were included in the final analysis and we selected all primary caregivers and their respective elder care recipients included in this subgroup analysis. Baseline survey data on 867 pairs of older persons and their primary caregivers were analyzed to determine the willingness of family caregivers to send their care recipients to a CIIC intermediary care center.

### 2.2. Measures

The structured questionnaires included the sociodemographic characteristics of older adults and primary family caregivers. Willingness to use a CIIC intermediary care service by caregivers was asked about by a prospective question with a simple “Yes” or “No” response: “If there is a short-term care service for the elder at the CIIC center in the community when you are not available, do you think you will use this service or not?”

#### CIIC Intermediary Care Service

The CIIC intermediary care service will provide respite care, which is formal care, but neither day care nor long-term institutionalized care. All the families assessed as eligible for intermediary care services will be invited to register for the CIIC center’s temporary respite care services, which is not part of the primary health care center of the sub-district. It is a totally free of charge service and the maximum length of stay is 10–14 days. The terms intermediary care, respite care, and short stay all refer to the CIIC intermediary care service in this study.

The baseline measures were organized by individual determinants for willingness to use CIIC stay in three different domains, approximating predisposing factors, enabling factors and need factors. Predisposing factors included age, gender, marital status, the relationship between the older person and caregiver, education, and occupation. They were categorized into dichotomous variables: age <60 years and ≥60 years, gender: male or female, marital status: currently married or not, education status: primary school finished or secondary school and above. The relationship between the older person and the caregiver were categorized as children, spouse, siblings, and others (relatives, friends, maids). Occupation included five types of job: no current job (i.e., caregiver is not currently working or no employment), own business, daily labor, private company staff, and government staff).

Enabling factors were assessed by the estimated monthly income, main income supporter for their family or not, and type of residence: original village residents or staying in a private housing estate. Estimated monthly income was categorized into income of less than or equal to 9000 baht, or more than 9000 baht.

Need factors of the care recipients; family caregiver burden; taking leave; quitting a job; getting sick due to taking care of elderly persons; having a secondary caregiver to back up the primary caregiver; underlying diseases such as hypertension, diabetes, and hyperlipidemia which were already confirmed and for which treatments had been given by health professionals; health behaviors such as smoking and drinking; and exercise habits of family caregivers were all explored. For smoking status, those who smoked any tobacco products (cigarettes, tobacco, or cheroot) either on some days or every day, were defined as current smokers. Former smokers or those who never smoked any tobacco products were categorized as current non-smokers. Consumption of any type of alcohol (spirit, beer, or wine) either on some days or every day were categorized as current alcohol drinkers and ex-alcoholics and non-drinkers were regarded as non-alcohol drinkers.

Care recipients’ demands were assessed by the Barthel’ index of activities of daily living (ADL). The ADL index is a standardized scale widely used by researchers and clinicians to assess the current level daily living activities of older adults. The ten fundamental daily activities included feeding, grooming, bathing, dressing, bowel and bladder care, toilet use, ambulation, transfer, and climbing stairs. It is commonly used by Thai researchers and validated in a Thai setting [25]. ADL total score ranged from 0 to 20, and it was categorized into two groups; 0–11 moderately to severely dependent, and ≥12 mildly dependent to independent elder participants.

Caregiver burden was assessed by Caregiver Burden Inventory (CBI) scoring. CBI is an internationally validated measurement tool to assess the impact of burden on different aspects of caregivers of older people [26,27]. It consists of five subscales: time dependence burden, measuring caregivers’ time restrictions and flexibility with time schedules (five items); developmental burden, evaluating the impact of failure to take opportunities (five items); physical burden, measuring physical impact of caregiving (four items); social burden, assessing social impacts (five items); and emotional burden, assessing feelings of embarrassment with elderly people (five items). Each item is scored by a 5-points Likert scale, ranging from 0 (not at all disruptive) to 4 (very disruptive) and each subscale ranges from 0 (low) to high (20). A physical burden subscale, including four items, was multiplied by 1.25 to make its range equivalent to other subscales. The total CBI subscale scores are summed up to get a 24-item total score. When the total score is higher than 24, it is considered to be an indicator of necessity to seek some form of respite care and when it is more than 36, it is considered as caregiver burnout. We categorized the total CBI score into dichotomous variables comprising a group with CBI total scores less than 24 and a group with a score of more than or equal to 24. 

We followed the WHO process of translation and adaptation of research instruments to translate all study instruments (ADL and CBI in this study) [28]. The overall reliability coefficients, Cronbach’s alpha of Barthel’s ADL and CBI were 0.90 and 0.77, respectively, after forward and backward translation, revision and editing after pilot testing by independent language experts and researchers.

### 2.3. Data Analysis

IBM SPSS version 22 (IBM Corporation, Armonk, NY, USA) was used for data analysis. Data were cleaned and recoding of some variables and computing the subscales and scales were performed as needed. Sociodemographic characteristics were analyzed by descriptive analysis. The dependent variable in this analysis was family caregivers’ willingness to send their older adults to a CIIC short stay center. Statistical significance of various factors was first measured through bivariate analysis (Chi-square test). Association of dependent and independent variables was analyzed via binary logistic regression. Statistical significance was defined as a *p* value < 0.05 with a 95% confidence interval (CI). 

## 3. Results

### 3.1. Sample Characteristics

**Figure 1:**
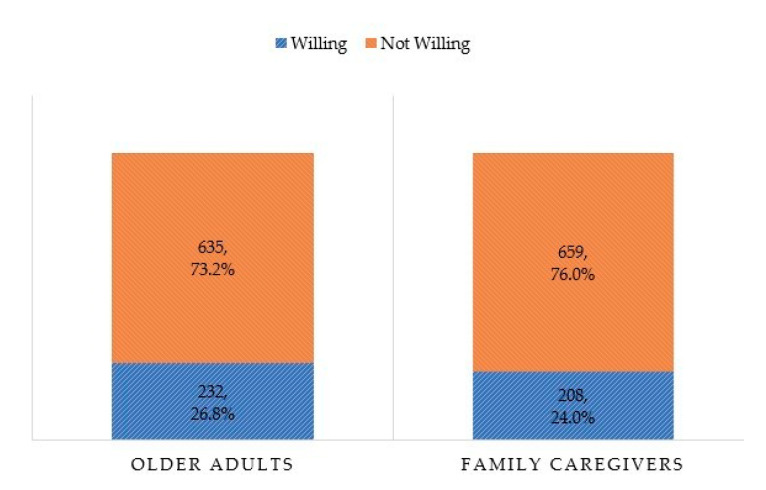
Willingness of study participants to use an intermediary care service at CIIC center, Maehia, Chiang Mai, Thailand 2019.

The baseline survey data for an intervention arm included a total of 867 pairs of older adults and their family caregivers. The mean ages of the care recipients and the caregivers were about 69.16 ± 8.33 and 55.27 ± 13.7 years, with females constituting 57.7% and 62.3%, respectively. Nearly three- quarters of them were residents from the original community (73.2%) and 26.8% of participants lived in housing estates where houses were separated in a private, gated community. Only 6.7% of housing estate residents were willing to use CIIC services and the preference for private care was the most common reason for reluctance to use such public services, followed by having back up caregivers, and being healthy with minimal care needs. Seventy percent of caregivers were married (70.5%), and 66.9% had completed secondary school and above. Currently working family caregivers (about 68.9%) and private business (29.2%) were the most common types of work, followed by daily labor (16.4%), private company staff (12.2%), and government staff (11.1%). Half were main income supporters of their families (49.9%) and 48.9% had estimated monthly income of more than 9000 baht. The two most common types of older persons’ family caregivers were children (son, daughter or grandchild) (46.8%) and spouses (44.8%). Regarding underlying diseases of family caregivers, hypertension (28.0%), diabetes (9.3%), and hyperlipidemia (8.2%) were noted. About 21.0% of study participants did not exercise, 27.7% drink alcohol currently and 8.8% were current smokers. The characteristics of the family caregivers are summarized in Table 1. About 26.8% of elderly participants and approximately a quarter of family caregivers (24.0%) were willing to use a short stay at a CIIC center temporarily, as shown in Figure 1.

### 3.2. The Burden on Family Caregivers

The burden on family caregivers was assessed by means of Caregiver Burden Inventory (CBI) scoring and other determinants with “Yes” or “No” responses such as taking leave, quitting jobs, getting sick while taking care of older persons, and care recipients’ demands were assessed by ADL total scores. The total score of each CBI subscale (time-dependence, physical, emotional, social, developmental) was calculated and burdens in each domain were as followed; time-dependence burden 25.7%, physical burden 21.2%, emotional burden 18.9%, social burden 15.9%, and developmental burden 15.1%. The time-dependence domain was reported to be the highest level of burden and about 5.5% of the participants had a total CBI score of more than 24, indicating a need for respite care. About 37.7% of caregivers did not have secondary caregivers to back up their caregiving and 4.6% of caregivers needed to take leave from their jobs frequently to take care of their older adults. The maximum frequency of leave was about 20 times in the previous year. The frequencies of family caregivers who got sick and quitted their jobs were 2.8% and 5.7%, respectively. The mean ADL total score of elderly participants was 19.08 ± 3.00. About 96.7% were mildly dependent to independent (ADL total scores more than or equal to 12) and 3.3% had moderate to severe dependency, as described in Table 2.

### 3.3. Factors Associated with the Family Caregivers’ Willingness to Send Older Adults to CIIC Intermediary Care Center

Approximately a quarter of family caregivers (24.0%) and 26.8% of elder participants were willing to use a short stay service at CIIC center temporarily. Care recipients’ demands did not affect their caregivers’ willingness for respite care, and we noted that the caregiver characteristics determined their willingness regarding a CIIC short stay. Type of caregivers affected willingness regarding respite care service, where we noted that spouse caregivers (Adj OR 2.47: 95% CI: 1.59–3.83) and others (relatives, maids, friends) (Adj OR 2.89: 95% CI: 1.08–7.66) were more willing to use the CIIC service than children. Those with family caregiver burden total scores of more than or equal to 24 (Adj OR 8.47: 95% CI: 3.71–19.34), having diabetes (Adj OR 3.53: 95% CI: 1.91–6.51), and those who needed to take leave from their job to take care of elder persons (Adj OR 3.52: 95% CI: 1.58–7.81), were more willing to receive CIIC services. Sociodemographic factors that affected their willingness to use CIIC short stay services were being original community residents (Adj OR 6.00: 95% CI: 3.32–10.84), monthly income ≤ 9000 baht (Adj OR 1.75: 95% CI: 1.05–2.90), with own business (Adj OR 2.43: 95% CI: 1.45–4.11), and working at private company (Adj OR 2.71: 95% CI: 1.23–5.97). Family caregivers’ age, gender, education, marital status, being main income supporter of the family and having secondary caregivers did not have any significant associations with willingness to send older adults to a CIIC intermediary care center, as described in Table 3.

## 4. Discussion

Our study explored the factors affecting the willingness of family caregivers to send their elder persons to a CIIC intermediary care center. We noted that the characteristics of family caregivers themselves determined their willingness towards using the CIIC respite care service, while care recipients’ demands on the family caregiver did not have any associations with this willingness. This may be due to the fact that the majority of older adults in our study were independent in terms of Barthel’s activity of daily living scoring, with only 3.3% being moderately to severely dependent. 

We analyzed the caregiver characteristics in three domains according to Anderson’ behavioral model of health services use. First of all, one of the predisposing factors, relationship between older persons and caregivers, was a major determinant of their willingness to use a short stay service, with spouse caregivers being 2.47 times and others (relatives, maids, friends) 2.89 times more likely to use the short stay service than children. As spouse caregivers were older, they were more likely to be burdened and to suffer burnout than younger children caregivers [29]. Moreover, children’s perception of moral obligation to support and take care of parents as a form of repayment is a common societal norm in Thai culture, and this might have negatively affected their willingness to send elder persons to a respite care center [24]. This finding was however, contradictory to another study in Vietnam where older adults were less willing to use elderly care services even when free of charge, regardless of whether they were staying with spouses or children [30]. The job demands and nature of the jobs also determined their burden and willingness to seek for respite care, as we noted that caregivers working at private companies and having their own business were more willing to use the CIIC service. A higher willingness of 2.71 times among private company workers and 2.43 times among private business supported this positive correlation between increased burden, opportunity costs, and increased willingness to use the CIIC service. 

Other predisposing factors of family caregivers’ age, gender, education, and marital status did not have any significant association with their willingness to use a CIIC stay. Although education status was one of the social determinants of health, with many scientific works confirming the relationship between level of education and caring for one’s own health along with health care service usage, our study did not find any significant association between education with willingness to use CIIC intermediary care service [31].

Enabling factors such as income and residential status were explored, and both had enabling effects on family caregivers using the intermediary care service for their care recipients. Generally, higher income or socioeconomic status is one of the enabling factors for willingness to use a paid respite care service [32]. However, being a free of charge respite care service located in the community, the CIIC short stay was easily accessible to lower income and higher burden family caregivers and this might have positively affected their willingness to use it. This finding was supported as caregivers earning less than or equal to 9000 baht per month were more willing to use the service than those earning more than 9000 baht per month.

Families residing in their original village communities were six times more likely to use the service than their counterparts residing in private housing estates in a gated community. Residents in the original village communities lived in traditional houses, in a mix of rich or poor households. Higher community bonding, sense of security and social safety might have positively affected their willingness to send their care recipients to a CIIC intermediary care center. This finding was different from another study in Thailand where the prevalence of household needs for caregiving was highest in the richest households [33]. Housing type is a proxy indicator of economic status and residents in housing estates are relatively wealthier than original village residents. As a result, housing estate residents, who are economically advantaged and keen to stay privately may select private and luxury facilities. Nonetheless, another study noted a similar finding of original community residents being more inclined to participate in a community group exercise program where they can share community resources, with a sense of social security in their environment [34].

In terms of need factors, family care giver burden reflected by CBI scoring, taking leave for caregiving, and caregiver’s physical health status were noted to be associated with willingness for CIIC stay. Family caregivers having CBI total scores of more than or equal to 24 were 8.47 times more inclined to use the CIIC service compared to their counterparts with CBI total scores <24. This finding was consistent with other studies where increased caregiver burden was one of the predictors of using residential respite services [35,36,37]. It can be inferred that the short-stay service can serve as a break for family caregivers when they are suffering heavily from burnout from providing care to their loved ones. Caregivers who had to take leave to care for elder persons in the past year, were also 3.5 times more likely to use the CIIC service compared to their counterparts. Such caregivers might have turned to the short stay service as a solution when they were no longer able to take leave anymore, or were devastated by their care recipient’s demands which had taken a toll on their health and economic status in the long run. 

The physical health of caregivers also determined their willingness to use the intermediary care service. In terms of underlying diseases, 9.3% of caregivers had diabetes and they were 3.5 times more inclined to use CIIC service compared to those without diabetes. Managing diabetes along with taking care of older persons may increase the burden which could explain this increased willingness for CIIC stay. Another study in Europe also noted the increased odds of outpatient visits among diabetic caregivers of Alzheimer’s disease sufferers, although they did not find any direct effect of diabetes status on caregiver burden [38]. This is an alarming feature as these caregivers will become elder care recipients in the near future and the burden of chronic diseases will add to the burden of caregiving and subsequent negative impacts on the family, community, and country. Other possible need factors such as having hypertension, hyperlipidemia, quitting a job due to caregiving, getting sick due to caregiving, and not having secondary caregivers did not have any significant association with willingness for CIIC stay.

There are some limitations of this study to be discussed. Due to the nature of a cross sectional survey, our results could not determine the causality of motivators when using a respite care service. Moreover, due to the small number of heavily dependent care recipients, our findings could not provide statistical associations with ADL. Therefore, the findings on caregiver characteristics affecting willingness for CIIC short stay cannot be generalized for caregivers with highly dependent care recipients. Another critical factor, duration of care, might have also had an impact on the willingness of intermediary care service use; however, this question was not included in this study.

## 5. Conclusions

Although caregivers may be reluctant to use an intermediary care service for cultural reasons, the findings of this study highlighted that the need for such a service is still evident. A formal care service in the form of a short stay for elder persons when family caregivers are temporarily unavailable or burned out is wanted by both care recipients and their caregivers. Such a service could prevent the likelihood of abuse and neglect. Moreover, early screening of dependency of older adults and of caregiver burden can detect minimal care needs, preventing heavy long-term care needs, which in turn would prevent family caregiver burnout and loss of productivity. Despite the vast majority of the study population being mildly dependent to independent older adults, the burden on family caregivers and their willingness to seek respite care service is significant. With an increasingly aging population and prevalence of NCDs, more willingness towards staying in a CIIC among diabetic caregivers was a crucial finding. Public health interventions along with health promotion activities should be targeted, modifiable, lifestyle-related risk factors, such as smoking or physical inactivity, to prevent or reduce not only NCDs but also caregiver burden subsequently. The finding of community support as an enabling factor for health care service usage, reflected by the higher willingness of original community residents to use the intermediary care, can also be important for implications in further studies in Thailand. We recommend that community-based respite care services are needed, and should be accessible to their local communities in order to strengthen the current traditional family based long-term care model in Thailand. 

## Figures and Tables

**Table 1:** Characteristics of the family caregivers, Maehia subdistrict, Chiang Mai, Thailand 2019 (*n* = 867).

Family Caregivers	Willingness to Use a CIIC Intermediary Care Service		
	Yes	No	Total
	*n* (%)	*n* (%)	*n* (%)
Age			
<60 years	98(47.1)	366(55.5)	464(53.5)
≥60 years	110(52.9)	293(44.5)	403(46.5)
Sex			
Male	70(33.7)	257(39.0)	327(37.7)
Female	138(66.3)	402(61.0)	540(62.3)
Residential type			
Housing estate	14(6.7)	218(33.1)	232(26.8)
Original community	194(93.3)	441(66.9)	635(73.2)
Marital status			
Married	152(73.1)	459(69.7)	611(70.5)
Not married (single, separated, divorced, widowed)	56(26.9)	200(30.3)	256(29.5)
Education			
Primary school completed	83(39.9)	204(31.0)	287(33.1)
Secondary school and above	125(60.1)	455(69.0)	580(66.9)
Occupation			
No current job	58(27.9)	212(32.2)	270(31.1)
Own business	77(37.0)	176(26.7)	253(29.2)
Daily labor	38(18.3)	104(15.7)	142(16.4)
Private company staff	20(9.6)	86(13.1)	106(12.2)
Government staff	15(7.2)	81(12.3)	96(11.1)
Estimated monthly income			
>9000 baht	88(42.3)	336(51.0)	424(48.9)
≤9000 baht	120(57.7)	323(49.0)	443(51.1)
Main income supporter of the family			
No	99(47.6)	335(50.8)	434(50.1)
Yes	109(52.4)	324(49.2)	433(49.9)
Relationship between older persons and caregivers			
Son, Daughter, Grand child	68(32.7)	338(51.3)	406(46.8)
Spouse	119(57.2)	269(40.8)	388(44.8)
Siblings	13(6.3)	34(5.2)	47(5.4)
Others (relatives, maids, friends)	8(3.8)	18(2.7)	26(3.0)
Current Smoking			
No	190(91.3)	601(91.2)	791(91.2)
Yes	18(8.7)	58(8.8)	76(8.8)
Current Alcohol drinking			
No	159(76.4)	468(71.0)	627(72.3)
Yes	49(23.6)	191(29.0)	240(27.7)
Exercise habit			
No Exercise	39(18.8)	143(21.7)	182(21.0)
Exercise but not regularly	144(69.2)	425(64.5)	569(65.6)
Exercise regularly	25(12.0)	91(13.8)	116(13.4)
Underlying diseases			
Diabetes			
No	167(80.3)	619(93.9)	786(90.7)
Yes	41(19.7)	40(6.1)	81(9.3)
Hypertension			
No	147(70.7)	477(72.4)	624(72.0)
Yes	61(29.3)	182(27.6)	243(28.0)
Hyperlipidemia			
No	189(90.9)	607(92.1)	796(91.8)
Yes	19(9.1)	52(7.9)	71(8.2)

**Table 2:** The burden of family caregivers, Maehia subdistrict, Chiang Mai, Thailand 2019.

The Burden of Family Caregiver	Willingness to Use a CIIC Intermediary Care Service		
	Yes	No	Total
	*n* (%)	*n* (%)	*n* (%)
Having secondary caregivers			
No	72 (34.6)	255 (38.7)	327 (37.7)
Yes	136 (65.4)	404 (61.3)	540 (62.3)
Need to take leaves for caregiving			
No	190 (91.3)	637 (96.7)	827 (95.4)
Yes	18 (8.7)	22 (3.3)	40 (4.6)
Need to quit the jobs for caregiving			
No	197 (94.7)	621 (94.2)	818 (94.3)
Yes	11 (5.3)	38 (5.8)	49 (5.7)
Got sick by caregiving			
No	201 (96.6)	642 (97.4)	843 (97.2)
Yes	7 (3.4)	17 (2.6)	24 (2.8)
CBI total scores			
<24	176 (84.6)	643 (97.6)	819 (94.5)
≥24	32 (15.4)	16 (2.4)	48 (5.5)
Care recipients’ demands according to ADL total scores			
Mildly dependent to independent ≥12	201 (96.6)	637 (96.7)	838 (96.7)
Moderately to severely dependent <12	7 (3.4)	22 (3.3)	29 (3.3)

CBI = 24 item Caregiver Burden Inventory score, ADL = Barthel’s Activity of Daily Living scores.

**Table 3:** Factors affecting the willingness of family caregivers to send their elderly to CIIC Intermediary care center, Maehia subdistrict, Chiang Mai, Thailand 2019.

	Willingness to Use a CIIC Intermediary Care Service				
	Frequency (%)	Adjusted OR	95% Confidence Interval	*p* Value	
Predisposing factors					
Age of family caregivers					
<60 years	98 (21.2)	Referent			
≥60 years	110 (27.3)	0.89	0.54–1.41		0.58
Gender of family caregivers					
Male	70 (21.4)	Referent			
Female	138 (25.6)	1.32	0.91–1.94		0.15
Relationship between older persons and caregivers					
Son, Daughter, Grand child	68 (16.7)	Referent			
Spouse	119 (30.7)	2.47 **	1.59–3.83		<0.01
Siblings	13 (27.7)	2.21	0.99–4.91		0.05
Others (relatives, maids, friends)	8 (30.8)	2.89 *	1.08–7.66		0.03
Education					
Primary school completed	83 (28.9)	Referent			
Secondary school and above	125 (60.1)	1.38	0.89–2.16		0.15
Occupation					
No current job	58 (21.5)	Referent			
Own business	77 (30.4)	2.43 **	1.45–4.11		<0.01
Daily labor	38 (26.8)	1.79	0.98–3.25		0.06
Private company staff	20 (18.9)	2.71 *	1.23–5.97		0.01
Government staff	15 (15.6)	1.35	0.60–3.07		0.47
Enabling factors					
Residential status					
Housing estate	14 (6.0)	Referent			
Original community	194 (30.6)	6.00 **	3.32–10.84		<0.01
Estimated monthly income					
>9000 baht	88 (20.8)	Referent			
≤9000 baht	120 (27.1)	1.75 *	1.05–2.90		0.03
Need factors					
Having secondary caregiver					
Yes	72 (21.9)	Referent			
No	136 (25.2)	0.91	0.63–1.33		0.63
Underlying diseases of family caregivers					
Diabetes					
No	167 (21.3)	Referent			
Yes	41 (50.6)	3.53 **	1.91–6.51		<0.01
Hypertension					
No	147 (23.6)	Referent			
Yes	61 (25.1)	0.67	0.42–1.07		0.09
Hyperlipidemia					
No	189 (23.7)	Referent			
Yes	19 (27.0)	1.18	0.60–2.32		0.63
Got sick by caregiving					
No	201 (23.8)	Referent			
Yes	7 (29.2)	0.37	0.11–1.30		0.12
Need to take leave for caregiving					
No	190 (23.0)	Referent			
Yes	18 (45.0)	3.52 **	1.58–7.81		<0.01
Need to quit the job for caregiving					
No	197 (24.1)	Referent			
Yes	11 (22.4)	0.71	0.34–1.52		0.38
CBI total scores of family caregivers					
CBI < 24	176 (21.5)	Referent			
CBI ≥ 24	32 (66.7)	8.47 **	3.71–19.34		<0.01
Care recipients’ demands by Barthel ADL total scores					
Mildly dependent to independent ≥12	201 (24.0)	Referent			
Moderately to severely dependent <12	7 (24.1)	0.57	0.18–1.76		0.33

ADL: Barthel’s Activity of Daily Living, CBI: 24 item Caregiver burden Inventory Scale, Adjusted OR: Adjusted odd ratio, * *p* value < 0.05, ** *p* value < 0.01.

## Data Availability

The data presented in this study are available on request from the corresponding author. The data are not publicly available because this study was a sub-group analysis of baseline data of the intervention clusters from a cluster randomized trial and the final analysis was ongoing and publications of the whole cluster randomized trial has not finished yet.
